# Estimating Bacterial Diversity for Ecological Studies: Methods, Metrics, and Assumptions

**DOI:** 10.1371/journal.pone.0125356

**Published:** 2015-04-27

**Authors:** Julia Birtel, Jean-Claude Walser, Samuel Pichon, Helmut Bürgmann, Blake Matthews

**Affiliations:** 1 Eawag, Department of Aquatic Ecology, Kastanienbaum, Switzerland; 2 Department of Environmental Systems Sciences (D-USYS), Swiss Federal Insitute of Technology (ETH), Zürich, Switzerland; 3 Genetic Diversity Centre (GDC), Department of Environmental System Sciences (D-USYS), Swiss Federal Institute of Technology (ETH), Zürich, Switzerland; 4 Department of Environmental Sciences, Zoology and Evolution, Universität Basel, Basel, Switzerland; 5 Eawag, Department of Surface Waters, Kastanienbaum, Switzerland; Argonne National Lab, UNITED STATES

## Abstract

Methods to estimate microbial diversity have developed rapidly in an effort to understand the distribution and diversity of microorganisms in natural environments. For bacterial communities, the 16S rRNA gene is the phylogenetic marker gene of choice, but most studies select only a specific region of the 16S rRNA to estimate bacterial diversity. Whereas biases derived from from DNA extraction, primer choice and PCR amplification are well documented, we here address how the choice of variable region can influence a wide range of standard ecological metrics, such as species richness, phylogenetic diversity, β-diversity and rank-abundance distributions. We have used Illumina paired-end sequencing to estimate the bacterial diversity of 20 natural lakes across Switzerland derived from three trimmed variable 16S rRNA regions (V3, V4, V5). Species richness, phylogenetic diversity, community composition, β-diversity, and rank-abundance distributions differed significantly between 16S rRNA regions. Overall, patterns of diversity quantified by the V3 and V5 regions were more similar to one another than those assessed by the V4 region. Similar results were obtained when analyzing the datasets with different sequence similarity thresholds used during sequences clustering and when the same analysis was used on a reference dataset of sequences from the Greengenes database. In addition we also measured species richness from the same lake samples using ARISA Fingerprinting, but did not find a strong relationship between species richness estimated by Illumina and ARISA. We conclude that the selection of 16S rRNA region significantly influences the estimation of bacterial diversity and species distributions and that caution is warranted when comparing data from different variable regions as well as when using different sequencing techniques.

## Introduction

**Fig 1:**
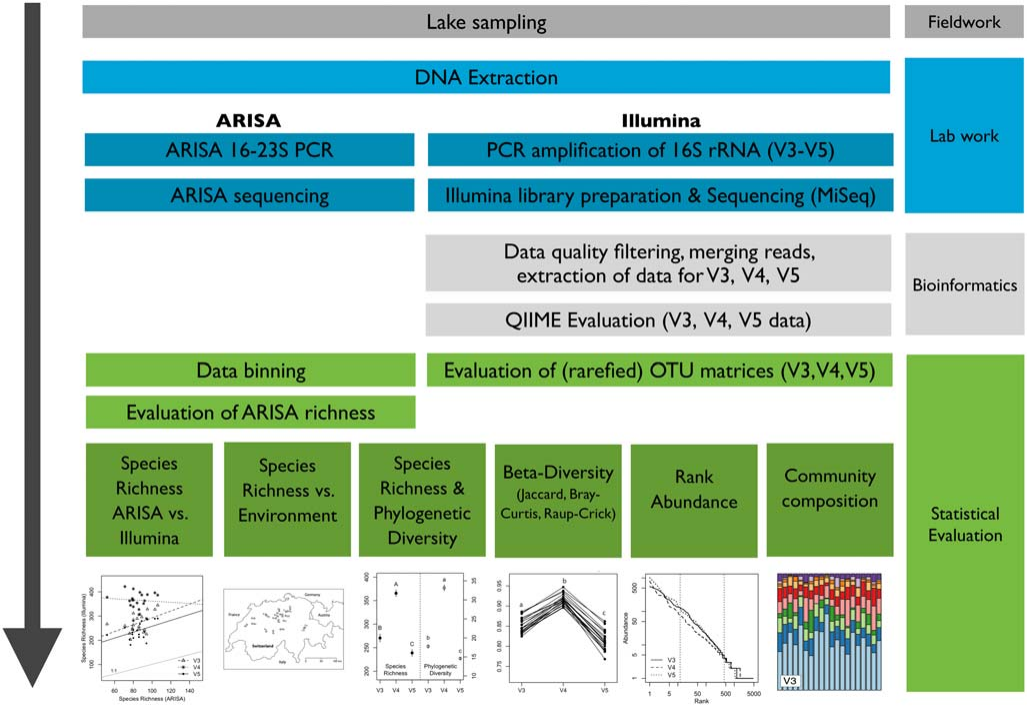
Conceptual figure of the study design. We have sampled 20 Swiss lakes, performed bioinformatical analyses and applied several ecological concepts on evaluating the microbial communities both with a fingerprinting method (ARISA), as well as by next generation sequencing (Illumina) from three variable regions of the 16S rRNA gene.

One of the central goals of microbial ecology is to measure and understand the distribution of diversity across spatial and temporal gradients. Ecologists are increasingly interested in using microbial communities to test a wide range of classic ecological hypotheses [1–4]. In the field of biogeography and macro-ecology, for example, microbial communities have been used in numerous comparative and experimental studies to test whether environmental properties could explain patterns of microbial diversity over a range of spatial scales [5–7]. In metacommunity ecology, there is a growing interest in the relative importance of dispersal and environmental conditions for explaining patterns of microbial diversity [8–10] and community assembly [11, 12]. Furthermore, in studies of biodiversity and ecosystem function [13], microbial communities are rapidly becoming model systems to explore how the composition (*i.e.* species richness and functional diversity) and abundance of microbial taxa can affect specific ecosystem functions and services [14–16]. Rank-abundance distributions of microbial communities have also been used to discriminate between alternate models of community assembly [11] and to understand how the rare biosphere might be functionally important [17, 18]. While the rapid development of methods to quantify microbial communities indeed shows great promise for testing ecological theory, it is increasingly important to evaluate how estimates of diversity vary due to technical and methodological considerations. Before the era of molecular techniques, microbial communities were commonly identified using microscopy or cultivation [19], but these methods are known for only capturing a fraction of the microbial taxa present in the environment [20]. Over the past three decades, microbial ecologists have increasingly been using the 16S rRNA [21, 22] as a marker gene to differentiate among microbial taxa, and the growing number of sequences in publicly accessible reference databases makes taxa identifications from 16S rRNA sequences more reliable. The characterization of microbial communities through 16S rRNA sequences has become a standard method in microbial ecology and a growing number of open-source sequence analysis tools (such as “mothur” [23], “QIIME” [24], or “RDP” [25]) facilitate the analysis of the large amount of sequences produced by modern massive parallel sequencing methods. Methods to characterize microbial communities through 16S rRNA sequences have developed rapidly. To reduce costs and time, the classic approach of creating clone banks [26, 27] followed by Sanger sequencing [28] has been replaced by next-generation sequencing (NGS) technologies [29] that produce huge amounts of sequences in very short amounts of time. This development has vastly increased our understanding of environmental microbial communities (*e.g.* [30]) and medically relevant microbiomes (*e.g.* [31, 32]). However, one of the drawbacks of NGS approaches is the limited read length and that sequencing the complete 16S rRNA gene of entire communities is still costly and methodologically complicated. NGS research is therefore commonly restricted to one or a few of the nine variable regions of the 16S rRNA gene. It is well known that different variable regions of the 16S rRNA gene vary in their abilities to identify and resolve microbial taxa [33–40], but there is no consensus about how to choose the best region to characterize microbial communities, and how robust a particular ecological conclusion is based on the choice of region. Here, using data from 20 bacterial community samples from Swiss lakes (S1 Fig), we focus on how the choice of variable region of the 16S rRNA gene influences common biodiversity metrics, including species richness (SR), community composition, phylogenetic diversity (PD) [42], the relationship between SR and PD and environmental gradients [41], *β*-Diversity [43] and rank-abundance distributions [44, 45] (Fig 1). We have used both Illumina MiSeq sequencing of the 16S rRNA gene between the V3 and V5 regions and a community Fingerprinting technique (ARISA = Applied automated Ribosomal Intergenic Spacer Analysis [46]), which uses the intergenic spacer region between the 16S and the 23S rRNA for determining bacterial diversity. We have used a set of natural lake samples to explore variation in the composition of the microbial communities, and to better understand how different variable regions of the 16S rRNA gene affect patterns of diversity, and furthermore applied the same analysis pipeline to reference data from the Greengenes database in order to compare our conclusions from natural samples to an existing database of sequences.

## Materials and Methods

### Ethics Statement

No permits were required to sample the lakes in this study. The authors also confirm that the sampling did not affect endangered or protected species.

### Sampling and DNA extraction

We sampled 20 Swiss lakes (S3 Table, S1 Fig) during the stratified period in the summer of 2011 (July to October). The lakes span a broad range of environmental characteristics, such as surface area, elevation, nutrient level, and dissolved organic carbon (DOC) concentrations. All lakes were sampled at their deepest point, using water samples integrated over the first five meters of the water column. Between 60–240 mL of lake water were filtered onto 0.2 *μ*m polyethersulfone filters (Supor 200 Membrane Disc Filters) at the same day of sampling, and filters were instantly frozen in liquid nitrogen and preserved at -80°C until further processing. Microbial DNA was extracted from preserved filters by enzymatic digestion and cetyltrimethyl ammonium bromide (CTAB) extraction [47]. The same DNA samples were used both for Automated Ribosomal Intergenic Spacer Analysis (ARISA) and NGS amplicon sequencing using Illumina technology.

### Amplicon sequencing

#### Sample preparations and sequencing

Using a high-fidelity polymerase (Phusion High-Fidelity PCR, New England Biolabs), we amplified the microbial 16S rRNA gene between the variable regions V3, V4, and V5 using a single primer set of custom-designed degenerate primers (forward primer: 327-ACACGGYCCARACTCCTAC-345, reverse primer: 969-TTGCWTCGAATTAAWCCAC-951). The primers were placed at conserved sites identified by Wang *et al.* [48] and designed to reduce primer-dimers and hairpin structures, and to reduce amplification of algal chloroplasts. To keep the PCR amplification bias low, we performed three low cycle PCR reactions (15 cycles) for each sample and subsequently pooled the PCR products. Pooled PCR products were then cleaned using AMPure XP beads (Beckman Coulter). Illumina library preparations of the amplified and cleaned PCR products were performed using the Nextera XT DNA Sample Preparation Kit (Illumina). The kit requires very low amounts of starting material (1ng) and uses dual-indexing, which allows the pooling of up to 96 samples in a single sequencing run. Paired-end (2×250nt) sequencing was performed on an Illumina MiSeq at the Genetic Diversity Centre (GDC) in Zurich.

#### Data processing

The raw Illumina reads were filtered and de-multiplexed using the Illumina MiSeq Reporter system software version 2.3. Overlapping reads were merged using SeqPrep [49], and possible mismatches between the overlapping fragments of the forward and reverse reads were corrected according to the base call with the higher sequencer-assigned quality score. Non-overlapping reads were kept separate. In a next step, the reads were quality-cleaned (minimum mean quality of 25) and size selected (minimum read length of 100nt) using PrinSeq Lite version 0.20.3 [49]. The quality filtering step also includes the clipping of ambiguous nucleotides from the ends and the removal of read with internal ambiguous nucleotides. Initial de-noising was performed with a 99% similarity clustering using USEARCH version 7.0.1001 [50]. As false priming cannot be completely excluded due to the degeneration of primers during PCR amplification of the 16S rRNA gene, de-noised reads were binned with the usearch option and the 16S reference database, both provided by QIIME (version 1.7.0, [24]). Reads without overlap were concatenated using Ns to facilitate read trimming. The binning parameters were determined from blasting subsets of the dataset against the NCBI 16S database to keep the error rate below 1%. *De novo* and reference chimera detection were performed with the UCHIME algorithm [50]. After these quality filtering steps, we retained a total of 112862 reads (merged and paired-end) from all lakes (average reads per lake sample: 5643; SD: 1155; S1 Table). The cleaned reads varied in size and coverage and were trimmed into subsets of fragments covering different parts of the targeted 16S rRNA gene region (V3, V4, V5, V3-V4, V4-V5). To trim the dataset into subset datasets we have used conserved 11-mer regions in proximity of each of the variable regions. The Gold 16S reference database was used to determine conserved 11-mer regions across species. The reads were screened based on the determined 11-mers and reads that did not carry the 11-mer were collected and the reverse complements of the those reads was screened again. In a next step, all reads that contained the 11-mer were aligned and trimmed to a specific length in order to cover the same variable region. 11-mer positions are included in S2 Table. Read lengths differed between subset-datasets and covered 120 nucleotides (nt) for the V3 and V4 dataset, 100 nt for V5 dataset, 360 nt for V3-V4 dataset and 311 nt for the V4-V5 dataset. Read counts for the subset datasets were in the range of hundreds to thousands of reads per lake sample (691–3085 reads) for V3, V4, and V5 and hundreds of reads per lake sample (184–432 reads) for the double-region datasets V3-V4 and V4-V5 (S1 Table). As the number of reads for sites covering two variable regions (V3-V4, V4-V5) was comparatively low compared to the number of reads in the single region datasets (S1 Table), we decided to perform most of the analyses using only the single region datasets. The Illumina sequences have been submitted to the Sequence Read Archive (SRA) and can be found under the project accession number SRP047505.

#### Data evaluation and statistical analysis

We used QIIME version 1.7.0 [24] for assigning operational taxonomic units (OTUs) at a sequence similarity of 97%. Briefly, we performed de novo OTU picking using usearch61 [50, 51] and picked a representative set of sequences. Taxonomy was assigned [52] using the most recent Greengenes database taxonomy (as of May 2013 [53]). To calculate phylogenetic diversity, OTUs were aligned and filtered, and a rooted tree was produced using the default fast tree option. QIIME analyses were performed separately for each region, as this allowed for de novo OTU picking and subsequent comparisons between the datasets of the different regions. After QIIME analyses, all subsequent analyses were performed in R (version 3.0.2, [54]). The R package ‘Phyloseq’ [55] was used to rarefy OTU tables to an even sampling depth. For analyses comparing datasets from single regions (V3, V4, and V5), datasets were rarefied to 650 reads, while for analyses of double region datasets (V3-V4 and V4-V5), datasets were rarefied to 150 reads per lake. The rarefaction was conducted to account for differences the number of reads in the datasets from the different regions, and most of the subsequent analyses were performed on the rarefied datasets. As the aim of our study was to compare the datasets of the three variable regions, we did not remove chloroplast sequences, which might affect diversity measures. Species richness (SR) was calculated as the number of unique OTUs, and phylogenetic diversity (PD) was calculated as the sum of phylogenetic branch lengths [56]. To minimize the influence of rarefaction on SR and PD, these measures were computed 1000 times, each time using a different rarefied OTU table, and then averaged. We used Analysis of variance (ANOVA) and a post-hoc Tukey HSD test to evaluate if SR and PD vary significantly between variable regions and major axis (MA) regressions [57] for comparing lake-specific SR and PD data between the different regions. Linear regressions were used to compare SR estimates from Illumina sequencing to ARISA estimates of SR of the 20 lakes and to test for relationships with environmental parameters. We quantified *β*-diversity of the V3, V4 and V5 region datasets using three different metrics: Jaccard (presence absence data), Bray-Curtis (abundance data) and Raup Crick (RC) [58]. The RC dissimilarity index is less sensitive to differences in SR among sites than the other dissimilarity metrics as it uses the total species pool of each dataset (*γ*-diversity) to calculate a null model distribution for each combination of samples (N = 10000 iterations), and the null distribution is then compared to the real number of shared species between samples to test wether lakes share more or less species than expected by chance. RC values range from -1 to 1, where a value of between -1 and -0.975 means that lakes are significantly more similar than expected by chance (*i.e.* lakes share more species than expected by the null model) and a value between +0.975 and +1 indicates that lakes are significantly more dissimilar than expected by the the null model (*i.e.* lakes share fewer species than expected by the null model). RC estimates between -0.975 and +0.975 indicate that there are no significant differences between the null model expectations and the observed number of shared species between lakes. Pairwise comparisons of RC between for the same combinations of lakes for each of the variable regions were plotted against each other to determine how the conclusions about microbial community similarity between lakes depend on the choice of variable region. Rank-abundance distributions were calculated in two ways. First, for a global comparison of abundance data, we combined the datasets for all 20 lakes and created rank-abundance tables for each of the single region datasets (V3, V4, V5). Second, for a pairwise comparison between lakes, the abundance data was ranked separately for each lake and region. We used the Kolmogorov-Smirnov (KS) test to test for significant differences in the shape of rank-abundance distributions between variable regions. In addition to describing bacterial diversity based on OTU abundances, we also investigated community composition at the class level from sequences clustered at 97% sequence similarity. We used paired t-tests to compare the relative abundance of the ten most common classes between communities defined by the V3, V4 and V5 datasets.

### ARISA Fingerprinting

For ARISA fingerprints of the microbial communities, the ribosomal intergenic spacer (ITS) region between the microbial 16S and 23S genes was amplified using a fluorescein (6-FAM)-labeled universal forward primer 1406f-6FAM and the bacteria-specific reverse primer 23Sr [60]. Binning of the ARISA peaks was performed in R 3.0.2 [54] using a window size of 1.5 and a shift value of 0.3 [61].

### Influence of sequence similarity threshold

A 97% sequence similarity threshold (SST) is often used during OTU picking to cluster similar sequences at species level [59]. We tested how different SST values (99, 95, 90, and 85% sequence similarity) affect SR, PD, *β*-diversity as well as rank-abundance distributions. For SR and PD calculations, we again performed 1000 repeated rarefactions and averaged the results to decrease the bias of random rarefaction.

### Reference dataset analysis

To test wether our observed differences among regions are generalizable beyond our lake survey, we ran our pipeline (Fig 1) with trimmed V3, V4 and V5 sequences from the Greengenes database (as of May 2013 [53]), which were trimmed in the same way as our lake survey samples. In order to make the comparison between the Greengenes dataset and our lake survey data, we randomly selected sequences in the same quantity as in our lake dataset (S3 Table). Subsequently, we performed the same QIIME analysis as described above and calculated SR and PD for the Greengenes database dataset, after rarefaction to the same level as the lake survey data (650 reads per lake sample).

## Results

### Species Richness and Phylogenetic Diversity

**Fig 2:**
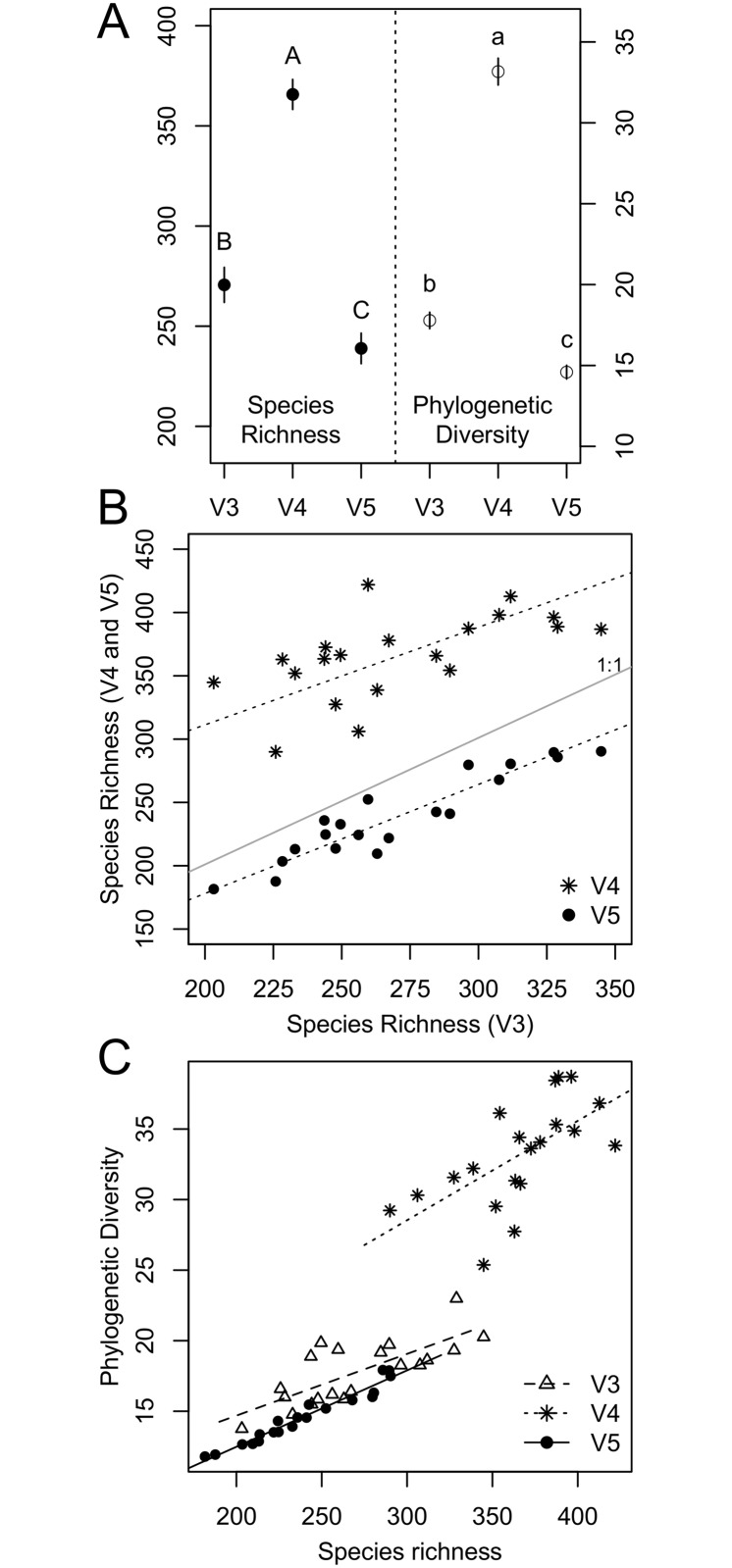
Number of observed species (SR; left side) and phylogenetic diversity (PD; right side) of the rarefied dataset from Illumina OTU data of the lake samples. A: SR and PD estimates for the three different regions. Points show the mean SR/PD of all lake samples and lines the standard error of the mean. B: SR of individual lakes from the V3 region plotted against SR of the same lake from the V4, respectively the V5 region dataset. The solid central line shows the 1-to-1 line, dashed lines show the Major Axis (MA) regressions of the two comparisons. C: SR (x-axis) plotted against PD (y-axis) for each of the three regions, where each dot represents one lake sample. The different symbols indicate the three different regions. Lines show the MA regression lines for each variable region dataset.

Absolute estimates of Illumina SR and PD varied significantly between the three variable regions for both rarefied (SR: F = 68.79 (p < 0.001), PD: F = 264.70 (p < 0.001); Fig 2A) and raw data (SR: F = 64.83 (p < 0.001), PD: F = 188.80 (p < 0.001); S2 Fig). To account for differences in read counts of the different lake datasets, which were generated by differences in sequencing depth, all of the following results are based on rarefied data only. The dataset based on the amplicons covering the V4 region resulted in significantly higher SR estimates (median: 366 unique OTUs) compared to the V3 (median: 262) and the V5 (median: 235) region. Corresponding with this, PD was also highest for the V4 region and significantly lower for both the V3 and the V5 region datasets (Fig 2A).

When comparing SR estimates of individual lakes between the different variable regions (Fig 2B; MA statistics: Table 1) we found that SR generated from the different variable regions were correlated. Even though absolute SR was higher for the V4 region datasets, the slopes of the relationship were neither different from one another, nor were they different from a slope of 1. When comparing the same patterns for PD, we found that the three regions resolve PD differentially (MA statistics: Table 1, S3 Fig). Together, these results suggest that V3 and V5 region datasets produce more similar patterns of diversity than V4 region dataset.

**Table 1:** Major axis (MA) regression results.

Figure	Rarefied (reads)	Parameter comparioson	x-axis	y-axis	R^2^	slope value	2.5% slope	97.5% slope	posthoc test
2B	Yes (650)	SR	V3	V4	0.351	0.771	0.337	1.518	a
2B	Yes (650)	SR	V3	V5	0.869	0.861	0.708	1.042	a
-	Yes (650)	SR	V4	V5	0.555	1.022	0.645	1.627	a
S2	Yes (650)	PD	V3	V4	0.583	1.872	1.273	3.063	a
S2	Yes (650)	PD	V3	V5	0.671	0.801	0.553	1.127	b
S2	Yes (650)	PD	V4	V5	0.799	2.099	1.664	2.758	a
2C	Yes (650)	SR vs. PD	V3 SR	V3 PD	0.568	22.855	15.954	40.228	a
2C	Yes (650)	SR vs. PD	V4 SR	V4 PD	0.400	14.197	8.822	36.001	a
2C	Yes (650)	SR vs. PD	V5 SR	V5 PD	0.951	18.466	16.600	20.803	a
-	No	SR	V3	V4	0.195	4.959	2.399	337.240	a
-	No	SR	V3	V5	0.926	0.783	0.679	0.898	b
-	No	SR	V4	V5	0.251	0.170	0.027	0.322	c
-	No	PD	V3	V4	0.435	3.318	2.073	7.300	a
-	No	PD	V3	V5	0.731	0.666	0.481	0.889	b
-	No	PD	V4	V5	0.515	4.565	3.056	8.652	a
-	No	SR vs. PD	V3 SR	V3 PD	0.624	26.520	19.156	43.051	a
-	No	SR vs. PD	V4 SR	V4 PD	0.824	24.030	19.554	31.156	a
-	No	SR vs. PD	V5 SR	V5 PD	0.960	23.996	21.783	26.708	a
S4	Yes (150)	SR	V4 SR	V3-V4 SR	0.305	1.393	0.664	3.753	a
S4	Yes (150)	SR	V4 SR	V3-V4 SR	0.212	1.870	0.795	12.044	a

MA was calculated in R using the lmodel2() function [77]. We used the 2.5% and 97.5% slope estimates to evaluate significant relationships between variables.

SR and PD were significantly positively correlated for all three regions (Fig 2C; MA statistics: Table 1), and the slopes of these relationships did not differ significantly between the different dataset. R^2^ estimates, however, were quite variable between datasets. This result indicates that SR and PD increase simultaneously, but also that the fit of the relationship between SR and PD strongly depends on the variable region (Table 1). We found a positive relationship when comparing SR estimates of the V4 region to SR estimates of the two datasets spanning over two variable regions (V3-V4 and V4-V5; Table 1, S4 Fig). The V4 region dataset showed higher estimates of SR compared to the extended regions, but the slopes of the relationship were not different from 1. Evaluating the relationships between SR, respectively PD, and environmental parameters, we found either weak or no relationships (S4 Table). The only significant (p < 0.05) relationships of environmental parameters and SR were detected were a negative correlation of SR of the V3 region dataset with PO_4_ concentrations (p = 0.02) and a positive correlation of SR of the V3 dataset with Chlorophyll a concentrations (p = 0.01). PD of the V4 region was negatively correlated with PO_4_ concentrations (p = 0.04) and positively correlated with Chlorophyll a concentrations (p = 0.02). Given these infrequent and weak relationships, it is unclear how the choice of variable region might alter our understanding of drivers of species diversity along environmental gradients.

### *β*-Diversity estimates

**Fig 3:**
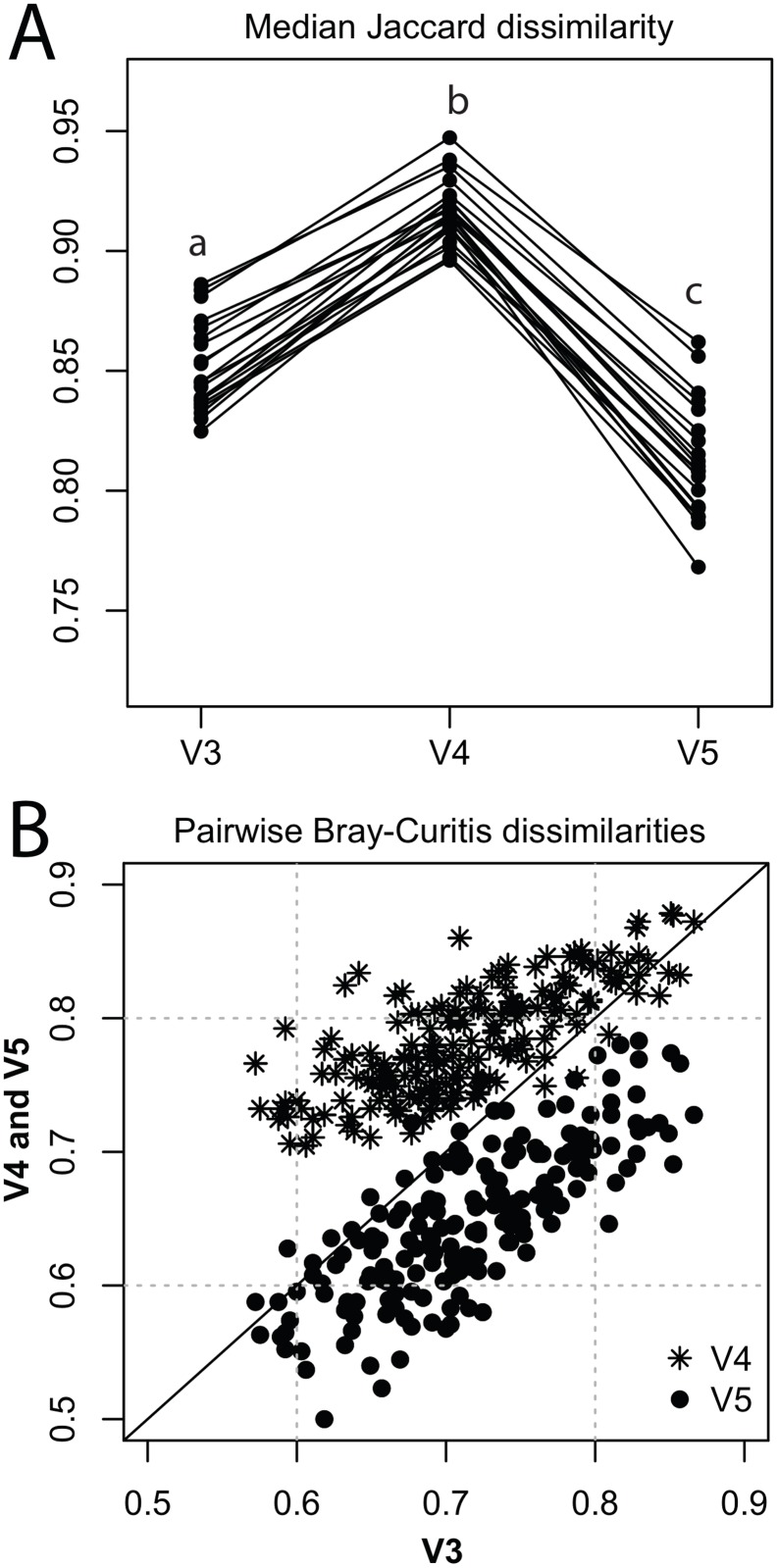
Comparison of Jaccard and Bray-Curtis dissimilarities between the variable 16S rRNA regions from the lake survey dataset. A: Median Jaccard dissimilarities of rarefied data for the three different variable regions. Each dot represents the median Jaccard dissimilarity from the pairwise comparisons of one lake to the other 19 lakes for one variable region. Lines connect the median dissimilarities of the same lake for the three different regions. B: Bray-Curtis pairwise dissimilarities of V3 plotted against V4 (stars) and V5 (circles) pairwise dissimilarities of the same pairwise combination of lakes. Central line shows 1:1 line.

**Fig 4:**
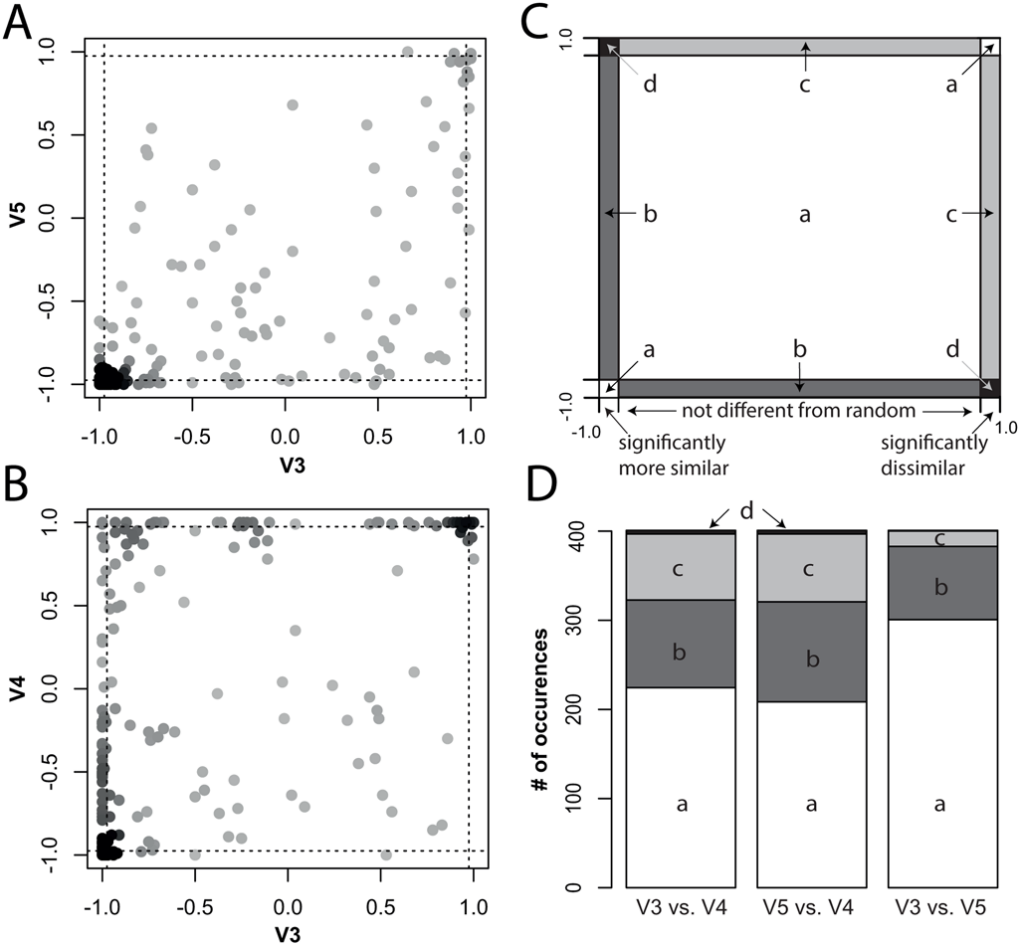
Raup-Crick (RC) comparisons between the three variable 16S rRNA regions from the lake survey dataset. A: Modified RC probability comparison of V3 and V4 (for rarefied data). Each dot represents the RC value of one pairwise dissimilarity comparison of the V3 region plotted against the same pairwise dissimilarity comparison of the V4 region. Values between -1 and -0.975 indicate that communities are significantly less dissimilar, and values between +0.975 and +1 that communities are significantly more dissimilar than expected by chance. Values between -0.975 and + 0.975 indicate that communities are not different from random expectation. Dashed lines show boundaries of significance (-0.975 and +0.975), where points falling between -1 and -0.975, respectively +0.975 and +1 indicate significant deviations from the null-model distribution. Dark areas in the plot represent high densities of points. B: Same as A, but for V3 plotted against V4 values. C: Conceptual figure illustrating the four different possible combinations when two RC-matrices are compared. a (white area): both regions come to the same conclusion about the dissimilarity among communities, b (dark grey): one of the regions estimates *β*-diversity of one lake pair to be significantly more similar than expected by chance while the other region estimates the *β*-diversity of the same lake pair to be not different from a random null-model distribution, c (light grey): one of the regions estimates *β*-diversity of one lake pair to be significantly more dissimilar than expected by chance while the other region estimates the *β*-diversity of the same lake pair to be not different from a random null-model distribution, d (black): cases where pairwise lake comparison of one region estimate *β*-diversity to be significantly more similar than expected by random chance, while the other region estimates *β*-diversity to be significantly more dissimilar than expected by chance. D: Barplot showing the number of cases where the compared regions come to the same (a) or different (b, c, d) conclusions about *β*-diversity. Coding is illustrated in panel C.

The bacterial lake communities appeared more dissimilar from each other when characterized by the V4 region, as indicated by Jaccard, Bray-Curtis, as well as Raup-Crick dissimilarities. Median Jaccard dissimilarities were significantly different between the three variable regions (F = 163.6, p < 0.001) and highest for the V4 dataset (Fig 3A). Bray-Curtis dissimilarities, which make use of abundance distributions, show a similar pattern, as pairwise dissimilarities between lakes were higher for the V4 dataset and lower for the V5 dataset as compared to the V3 dataset (Fig 3B). However, as both Jaccard and Bray-Curtis dissimilarities can be biased by differences in species richness among sites and regions, we also compared Raup-Crick estimates between the regions (Fig 4). The RC comparison revealed that both for the V3 and V5 region datasets, bacterial communities were on average more similar to each other than expected by random chance (Fig 4A). On the other hand, there was a high proportion of pairwise RC comparisons in which lakes shared fewer species than expected by the null model for the V4 region (Fig 4B). These patterns appear to be correlated for the V3 and V5 region, as, when plotted against each other, most points fall into the lower left corner (Fig 4A). This suggests that these two regions yield similar conclusions about community dissimilarity. However, when we compare RC for V3 and V4, there is more uncertainty in our conclusions. For example, more points fall at the edges of the plot (Fig 4B), indicating that one of the variable regions suggests the communities are not different from random expectations, while the other suggests the communities are either more or less dissimilar than expected by chance. Overall, pairwise comparisons of RC estimates indicate that the V3 and V5 region yield similar conclusions in a high proportion of pairwise lake comparisons (Fig 4D) and would lead to the conclusion that communities are more similar to each other than expected by random chance. Pairwise RC estimates of the microbial communities using the V4 region, however, less often come to the same conclusion as the other two variable regions and indicate that lakes are more dissimilar than expected by chance. These results indicate that higher SR estimates by the V4 region dataset also affects pairwise comparisons between communities and results in fewer shared species, as compared to the number of shared species found when comparing reads of the V3 or V5 region.

### Rank-abundance distributions

**Fig 5:**
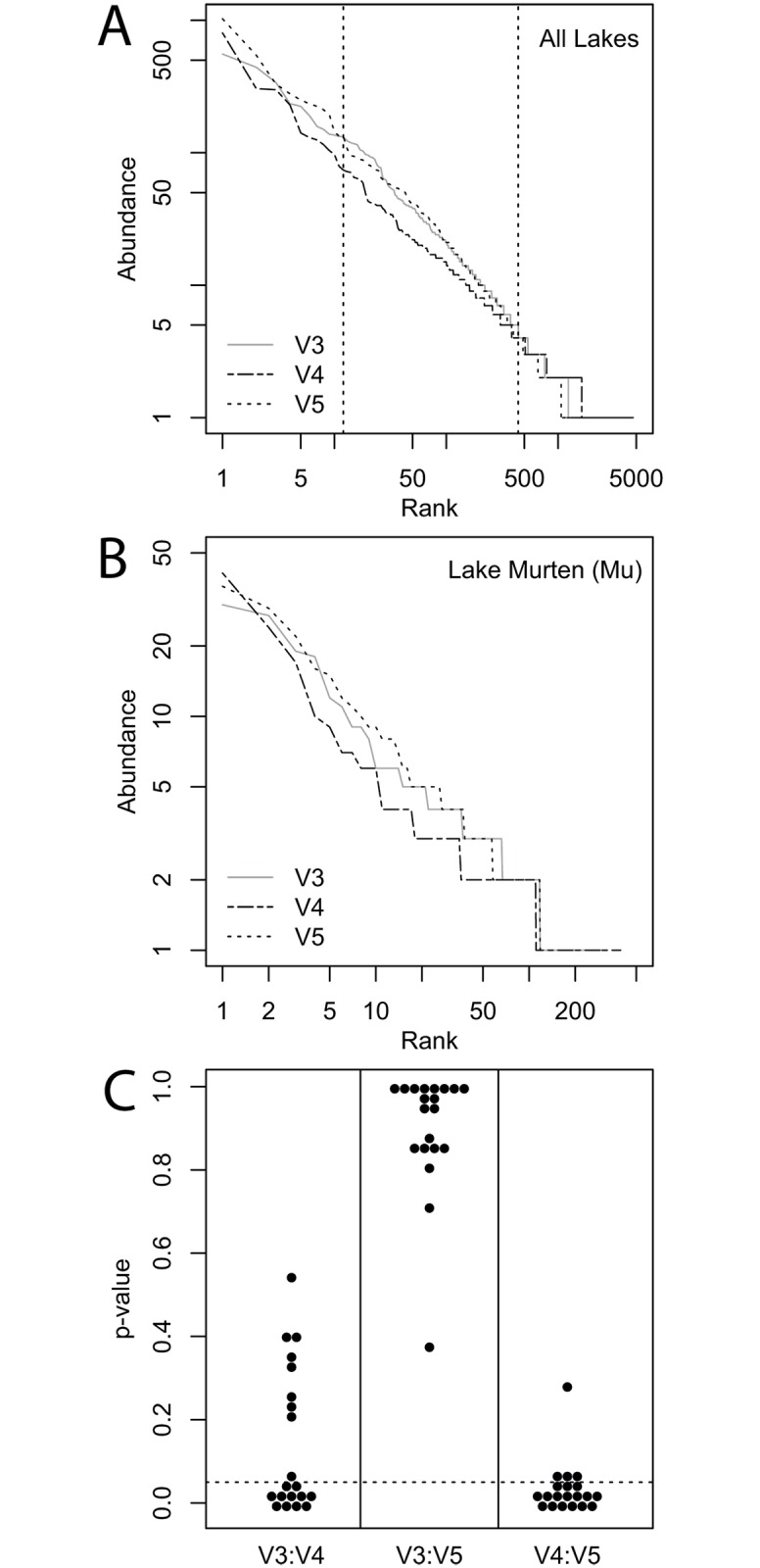
Rank-abundance evaluation of the variable 16S rRNA regions from the lake survey dataset. A: Rank-abundance plot of the complete dataset for each of the three variable regions, where abundance data was added up for all of the 20 lakes, plotted on log-log scale. Vertical dashed lines show the range of the rank-abundance plot (ranks 12 to 440) for which we found a significant difference between the rank-abundance distributions of V4 to V3 and V5. For the same region, the V3 and V5 rank-abundance distributions did not differ significantly from each other (significant Kolomogorov-Smirnov (KS) test: p < 0.05). B: Example rank-abundance plot of the rarefied data for one lake (Murtensee), plotted on log-log scale. X-axis: OTU rank, y-axis: OTU abundance. C: Result of KS-test using rank-abundance data of the individual lakes. X-axis: compared regions, y-axis: p-value distribution of KS test, dashed line plotted at p-value of 0.05. Each dot represents the comparison of rank-abundance curves from two regions of the same lake. P-values below 0.05 indicate a significant difference between the the rank-abundance distributions, whereas p-values above 0.05 indicate that there are no significant differences between two rank-abundance distributions.

The rank-abundance distributions of the three different variable regions also indicate significant differences between the bacterial communities characterized by the V4 dataset as compared to the V3 and V5 datasets. We detected significant differences in the shape of the rank-abundance distributions of the three variable regions, both when comparing individual lakes and when averaging over all lakes. Rank-abundance distributions averaged over all lakes were significantly different between the V4 and both the V3 and V5 region, but not between the V3 and V5 region datasets (Fig 5A). Comparing rank-abundance distributions of individual lakes (Fig 5B), we found no significant differences between the distributions of the V3 and the V5 datasets for any of the lakes, while for 55% of the lakes, significant differences between the distributions of the V3 and V4 datasets were detected, and for 80% of the lakes, the V4 rank-abundance distribution was significantly different from the distribution of V5 dataset (Fig 5C).

### Species richness comparisons of ARISA and Illumina sequencing data

**Fig 6:**
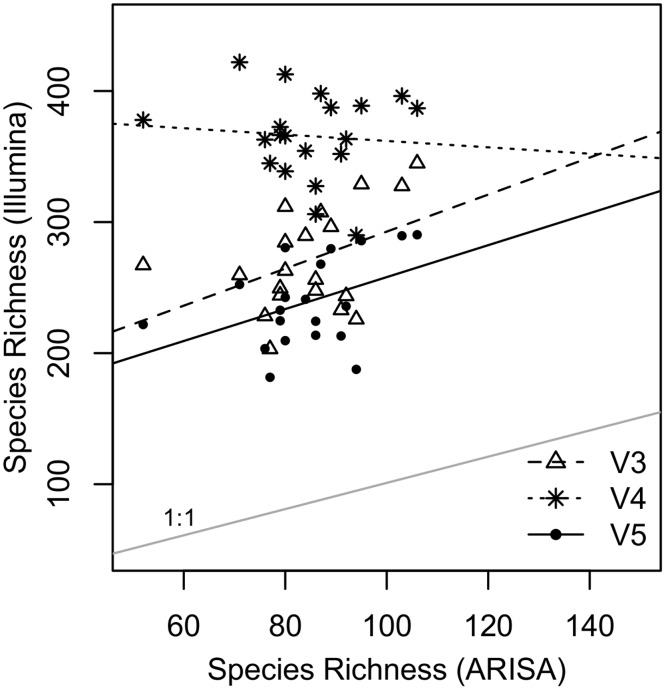
Species richness (SR) estimates from ARISA Fingerprinting plotted against SR estimates from Illumina sequencing. Each symbol represent the SR estimates of one lake for the two different methods clustered at a SST of 97%. Different symbols represent Illumina estimates from the three different regions. Lines show major linear regressions for each variable region (regression slopes: S5 Table).

Overall, the SR estimates based on the ARISA and the Illumina data did not show a significant (p < 0.05) correlation for any of the three variable regions (Fig 6, S5 Table), but SR of V3 and V4 from Illumina sequencing were marginally positively correlated with ARISA SR estimates (V3: F = 3.99, p = 0.06, slope = 1.42; V5: F = 3.89, p = 0.06, slope = 1.22), while no such correlation for V4 Illumina SR estimates and ARISA estimates was found (V4: F = 0.14, p = 0.72, slope = -0.25). The slopes of the individual 16S rRNA regions did not differ significantly from one another (F = 1.59, p = 0.21). Again, however, the V3 and V5 datasets appear more similar to each other as compared to the V4 dataset.

### Influence of sequence similarity threshold (SST)

We furthermore analyzed how the SST, which is used during the sequence analysis to cluster similar sequences, affects the above described indices and patterns (S5 Fig). As expected, SR and PD decreased for all regions when the SST was lowered, but SR and PD decreased at unequal rates. At levels between 90–95% SST, SR of the V4 region reached similar levels as SR of the V3 and V5 region at a SST of 97% (S5A Fig). SR estimates of V4 became very similar to SR estimates of V3 when the SST was lowered, while V5 SR remained lowest. PD, on the other hand, decreased at a much lower rate when the SST was reduced (S5B Fig). Even at a SST of 85%, PD of the V4 dataset was still approximately 2-fold higher than PD of the V3 and V5 dataset. In either case, the widening gap between the V4 region dataset and the V3 and V5 region datasets with increasing SST indicates that the differences between these regions are related to an increased diversity at higher clustering thresholds. As expected, Jaccard and Bray-Curtis mean pairwise dissimilarities also decreased, showing that the communities are more similar at a lower SST due to less stringent clustering parameters. Using presence-absence data (Jaccard dissimilarity), relative differences between mean pairwise dissimilarities of the three variable regions remained equal (S5C Fig), while when using abundance data (Bray-Curtis dissimilarity), differences between the datasets from the three variable regions became less pronounced and V3 and V4 dissimilarities converged (S5D Fig). Lowering the SST decreased the number of rare species and flattened rank-abundance distributions, resulting in steeper slopes of the rank-abundance curves for all three regions (S5E Fig). With a lowered SST, the V4 rank-abundance distribution became less different from the V3 distributions, but stayed significantly different from the V5 rank-abundance distributions for 30% of the lakes even at a SST of 85% (Kolgorov-Smirnov Test: p < 0.05; S6 Fig). Linear model comparisons of SR estimates from Illumina and ARISA overall revealed several cases where Illumina estimates of SR of the V3, as well as the V5 region, were marginally (p-value between 0.07 and 0.05) correlated with ARISA SR estimates (S5F Fig, S5 Table). SR estimates of the V4 region, however, never showed significant correlations with ARISA estimates of SR, irrespective of the SST value. Overall, the SST analysis revealed that differences between the three variable regions remain even when the sequences are clustered at different similarity thresholds.

### Taxonomic evaluation

**Fig 7:**
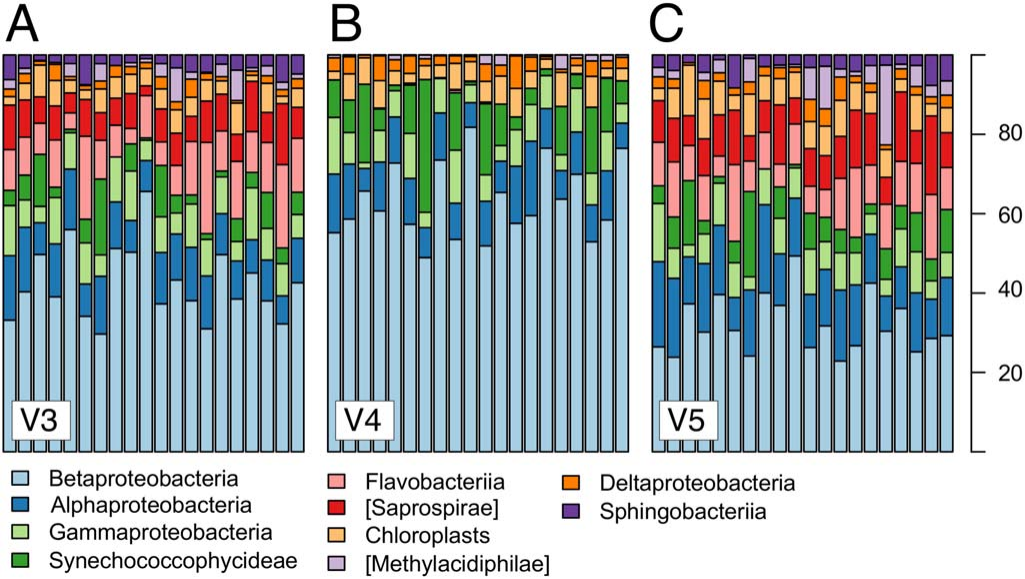
Barchart of the most abundant bacterial classes. Relative abundances of the ten most abundant bacterial classes across the V3, V4 and V5 datasets. Each bar represents the relative class distribution in one lake and each group of bars represents the relative abundances for one of the tree variable regions (V3, V4, V5). Bars are ordered from left to right by alphabetical order (see S1 Fig and S3 Table for more information about the lakes). Appendant results of paired t-test statistics are shown in S5 Table. Square brackets indicate candidate class names.

Analyzing the community composition based on bacterial classes of the V3, V4 and V5 region datasets revealed significant differences in relative abundances of the ten most abundant classes (Fig 7, S6 Table). Clearly, the relative abundances of the V3 and V5 datasets are more similar in their composition, while both of them differ markedly from the V4 dataset (Fig 7), and this observation is well supported by paired t-tests (S6 Table).

### Comparison with reference data

By analyzing the Greengenes dataset using the same parameters that were applied to the lake survey data, we detected that the V4 region consistently resolved PD differently than the V3 and V5 region datasets, but did not always show a higher SR (S7 Fig). Furthermore, the coupling of SR and PD was different for the reference data as compared to the lake survey data (S7 Fig). The slopes of the relationship varied significantly between variable regions (F = 25.88, p < 0.001). This result indicates that even when using a random selection of reference sequences, the V4 region resolves PD significantly higher than the V3 and V5 region.

## Discussion

In this study, we amplified the V3 to V5 regions of the microbial 16S rRNA from 20 natural lake water samples using a single bacteria-specific primer set, and after Illumina sequencing, trimmed the data into three datasets corresponding to three variable 16S rRNA regions (V3, V4, V5). We then estimated bacterial diversity and were able to show that the choice of variable region strongly influences the estimation of diversity based on SR and PD, and, as such, may significantly alter the ecological conclusions for a given study.

Most studies that investigated the influence of 16S rRNA region on diversity estimates have focussed on measures of *α*-diversity (*e.g.* [33, 48, 62–64]), whereas here, we additionally investigated the effect of 16S rRNA region on PD, community composition, rank-abundance distributions as well as *β*-diversity. One novel aspect of our approach is the use of PCR products produced by a single primer pair instead of using separate primer pairs for each of the variable 16S rRNA regions. This method minimizes the influence of different primer pairs on the composition of the PCR products, which can affect species composition and SR estimates [65]. Furthermore, we used the same bioinformatic pipeline for analyzing an environmental dataset originating from natural lake samples, as well as an *in-silico* dataset extracted from the Greengenes reference database. Comparing our dataset of environmental samples to a reference dataset allowed us to investigate the generality of differences between the variable regions of the 16S rRNA gene. Comparisons to other available datasets, however, would be necessary to show if this is a general pattern across reference data. We are aware that various choices made during sequence analysis, such as the selection of the reference database, the taxon calling, and the sequence clustering can influence our results, but we tried to minimize these results by carefully choosing our analysis pathway, by comparing our lake survey data to reference data, and by analyzing how the sequence clustering threshold influences various of the chosen measures of diversity (Fig 1).

The results from our lake survey dataset suggest that different variable regions of the 16S rRNA gene resolve SR and PD differently. Both SR and PD were significantly higher for the V4 region dataset, but we think that these parallel patterns for absolute estimates of SR and PD do not only arise from the fact that higher SR was measured for the V4 dataset, as PD estimates are not directly linked to the number of species in a system [66]. Instead, the results suggest that the three variable regions differ in how species composition and identities are resolved and contain different types of phylogenetic information. This finding was underlined by the fact that we found significant differences between the relative abundances of bacterial classes (Fig 7). The significantly higher estimates of PD for the V4 region were also found in the Greengenes reference dataset and were robust to changes in the sequence similarity threshold (SST) during sequence clustering (S5 Fig), suggesting that the patterns we obtained are inherent to the 16S regions themselves and not specific to our samples or to the OTU clustering threshold. Kim *et al.* [33] already suggested that the threshold for defining a molecular species must be adapted for variable regions of the 16S rRNA gene, and that it may be necessary to change SST depending on which of the nine 16S rRNA regions is chosen. Our lake survey results are in accordance with previous studies that have also found higher SR for the V4 region compared to the V3 and V5 regions [36, 62]. Vinje *et al.* [36] revealed that the V4 region contains in proportion at least twice as many informative sites compared to V3 and V5 to discriminate taxa, but they also noted that half of the discriminative sites were found outside of variable regions. Targeting the amplification of fragments where the number of discriminative sites is optimized would allow robust downstream analyses such as taxonomic assignment, phylogeny and species richness estimates. As a consequence, it is promising that improvements on read length are advancing for Illumina sequencing, as this will furthermore improve downstream analyses. Although the absolute number of identified species depended strongly on the region, estimates of SR derived from different 16S regions were highly correlated (Fig 2B). This is encouraging as it suggests that studies choosing different target regions may be comparable on a relative scale. However, this was not the case for PD, which seems to be strongly influenced by the choice of variable region. Furthermore, SR and PD were not correlated equally between the three regions.

Estimates of *β*-diversity (*i.e.* differences in diversity between the lake samples) were also particularly sensitive to the choice of variable region (Figs 3 and 4). Pairwise *β*-diversity dissimilarities based on Jaccard and Bray-Curtis were higher for the V4 dataset and lower for the V5 dataset as compared to the V3 dataset (Fig 3). By using the Raup-Crick (RC) dissimilarity matrix in addition to Jaccard and Bray-Curtis dissimilarities, we found that the differences in dissimilarities between the three regions are not only driven by the absolute number of species. RC matrices of the V3 and V5 region appear to be more similar to each other, as pairwise distance matrices show strong overlap. As a result, the V3 and V5 regions would yield similar conclusions about patterns of *β*-diversity, but using the V4 region could lead to dramatically different conclusions. In many pairwise comparisons between lakes, communities that were more similar than random expectation when using the V3 or V5 region were actually more dissimilar than expected when using the V4 region dataset (Fig 4D). Hence, the ecological conclusion is reversed based on the choice of region. One potential explanation for such results is that the V4 region resolves reads at different taxonomic levels than both the V3 and V5 regions, which leads to comparably less overlap between the communities and greater dissimilarities when comparing lake pairs. Interestingly, this may also happen when taking longer regions of the 16S rRNA into account. Longer regions should provide a better phylogenetic placement of an individual read, but they can still mask sample-to-sample differences depending on the similarity cutoff used for species definitions. Hence, comparing patterns of *β*-diversity among samples or studies (such as performed *e.g.* by Shade *et al.* [67]) will be sensitive to the choice of region of the 16S rRNA.

We also found that rank-abundance distributions can be significantly different depending on which variable region is analyzed (Fig 5). It is well known that rank-abundance distributions can be highly influenced by PCR artifacts [68] and sequencing errors [69], but as far as we are aware, the influence of the variable region of the 16S rRNA has not been investigated. The rank-abundance distributions of individual lakes using the V3 and V5 datasets were never significantly different from each other, while they were both significantly different to the distribution of the V4 region dataset for the majority of lakes. Rank-abundance curves are influenced by the way species abundances are distributed between the different taxa, and so the difference of the V4 rank-abundance curves is likely due to higher species richness in the V4 datasets. By decreasing the SST, we were able to show that the steepness of the rank-abundance curve increases, which indicates that species with low frequencies are lost as OTUs are clustered at less stringent SSTs.

While Pilloni *et al.* [75] demonstrated a strong correlation between NGS (454) and Fingerprinting (T-RFLP) data, we did not find the same pattern for the comparison between Illumina and ARISA. ARISA estimates SR by measuring the variability within the intergenic spacer region between the 16S and the 23S rRNA genes, whereas most NGS surveys target parts of the 16S rRNA to estimate SR. Generally, NGS is considered to be the more accurate technique for measuring microbial diversity, as there is an ever growing number of reference sequences to which NGS data can directly be compared. However, to our knowledge, the assumption that Illumina is a more appropriate method to estimate SR has not been tested intensively and few studies have directly compared the outcome of NGS richness to richness estimates from Fingerprinting techniques. A recent study [76] has compared ARISA and 454 Sequencing results and found strong correlations between richness estimates of ARISA and 454 sequencing, however, it is unknown how this relationship is influenced by the selected region of the 16S rRNA. Future work should investigate more in depth whether NGS sequencing and classical Fingerprinting techniques provide similar information about microbial diversity and evaluate how the variable region might affect the results.

Overall, our results suggest that the choice of variable region of the 16S rRNA might be important for many ecological studies, particularly in the context of biogeography [70], metacommunity theory [71, 72] or (human) microbiome studies [31, 73, 74], where information from diversity indices and rank-abundance distributions are common tools for comparing microbial communities. Currently, there is a lot of variation in the 16S rRNA regions used by different projects. Furthermore, the lack of a relationship between ARISA results and the three variable regions from Illumina sequencing also suggests that caution is warranted for comparing conclusions among studies which have used different techniques. Large scale projects, such as the Human Microbiome Project (HMP) or the Earth Microbiome project (EMP) try to make their data comparable by mostly sequencing the same part of the 16S rRNA, but many smaller studies use various parts of the 16S rRNA and thus make data comparisons between studies difficult or even impossible. We can not make general recommendations about which regions to use for NGS sequencing, but we have demonstrated, using three different variable 16S rRNA regions, that there are inherent differences between the regions of the 16S rRNA, which researchers should be aware of. This could motivate further research in order to find better techniques or approaches for estimating bacterial diversity, which we hope will lead to an improved understanding of bacterial communities.

## Supporting Information

S1 Fig Map of the sampling locations of the 20 Swiss lakes included in the lake survey as well as lake name abbreviations (see S1 Table). (TIF)

S2 Fig Species richness (SR) and phylogenetic diversity (PD) of the Illumina OTU data for the three different regions prior to rarefaction. Points show the mean and lines the standard error of the mean.
(TIF)

S3 Fig Rarefied PD of individual lakes from the V3 region plotted against PD of the same lake from V4, respectively V5. Central line shows 1:1 line, dashed lines show the Major Axis (MA) model regression slopes of the two comparisons.
(TIF)

S4 Fig SR of individual lakes from the V4 region plotted against SR of the same lake from the V3-V4, respectively the V4-V5 region dataset using data rarefied to 150 OTUs per sample. Central line shows 1:1 line, dashed lines show the MA model regression slopes of the comparisons.
(TIF)

S5 Fig Results from applying different sequence similarity threshold levels (SST) during OTU clustering (85, 90, 95, 97, and 99% sequence similarity). A: changes in SR, B: changes in PD, C: changes in Jaccard dissimilarities, D: changes in Bray-Curtis dissimilarities, E: changes in global rank-abundance slopes, F: changes in the linear model slopes between ARISA Fingerprints and Illumina sequencing. The graph shows the mean and standard error (SE) for each region and SST.
(TIF)

S6 Fig Changes in rank-abundance distributions of the three variable region datasets clustered at different sequence similarity threshold levels (85, 90, 95, 97, and 99% sequence similarity). Large plots: Rank plotted against abundance on a log-log scale. Small plots show the changes in significant differences between the three variable regions (see Fig 5C for a more detailed description of the inlay plots).
(TIF)

S7 Fig Analysis of SR and PD of the Greengenes reference dataset. A: Rarefied SR and PD calculated from a subset of the Greengenes database using the same parameters used as for the lake survey data. Points show the mean and lines the standard error of the mean. PD was significantly higher in the V4 dataset (F = 1406, p < 0.001) compared to the V3 and V5 datasets. The SR of the V4 region dataset was only significantly different form the V5, but not from the V3 dataset (F = 14.99, p < 0.001). B: SR plotted against PD for each of the three variable regions where each dot represents one lake sample. Lines show the MA regression model for each dataset. R^2^ values: V3 = 0.10, V4 = 0.49, V5 = 0.50.
(TIF)

S1 Table Number of quality filtered reads of the different lakes and variable regions of the 16S rRNA (untrimmed and trimmed reads). (PDF)

S2 Table Positions of 11-mers used for subsetting the quality filtered Illumina reads as well as the number of nucleotides (nt) of the trimmed datasets. Positions are given relative to E. coli 16S rRNA positions.
(PDF)

S3 Table Environmental parameters of the lakes that were sampled for this study. (PDF)

S4 Table Linear model statistics of species richness (SR; ARISA and Illumina sequencing) and phylogenetic diversity (PD) versus environmental parameters. (PDF)

S5 Table Linear model statistics of species richness (SR) estimates from ARISA and Illumina sequencing at different sequence similarity threshold (SST) clustering values. (PDF)

S6 Table Paired t-test results of the ten most abundant bacterial classes. (PDF)
