# Supplementary figures and images for: Estimating Bacterial Diversity for Ecological Studies: Methods, Metrics, and Assumptions
Source: PLoS One. 2015 Apr 27;10(4):e0125356. doi: 10.1371/journal.pone.0125356 (PMC4411174; doi:10.1371/journal.pone.0125356)

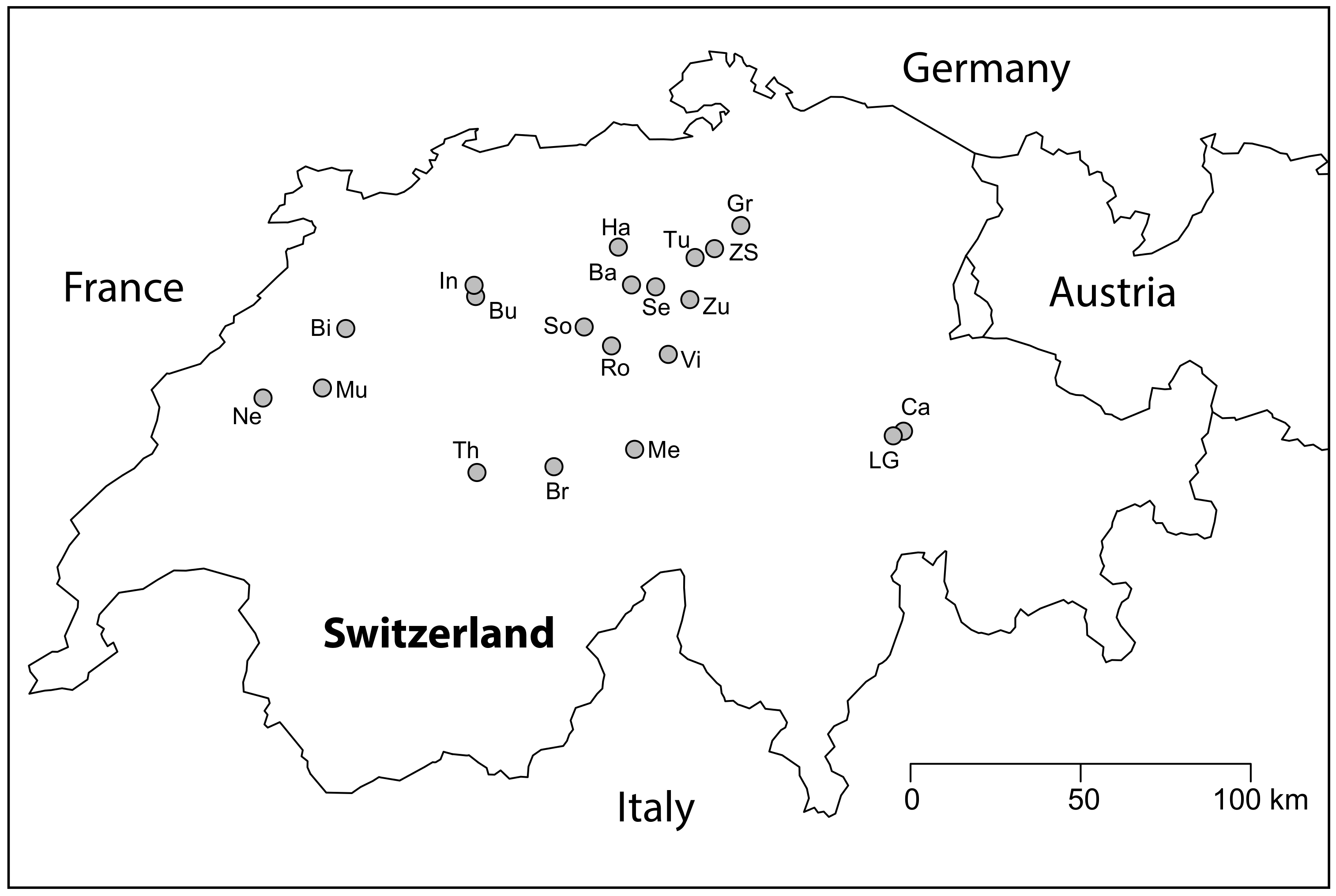

Supplement: S1 Fig — (TIF) [file pone.0125356.s001.tif]

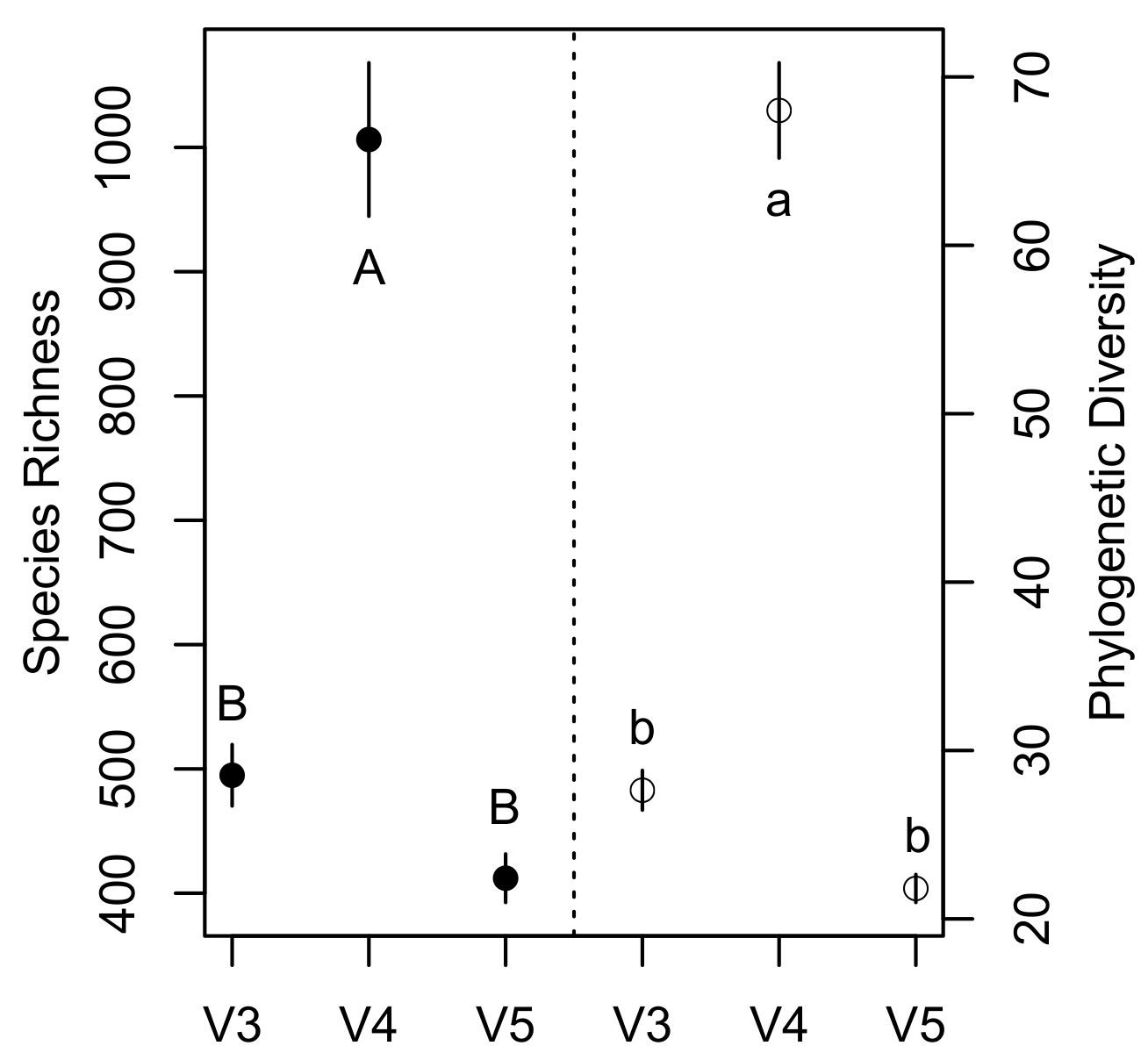

Supplement: S2 Fig — Points show the mean and lines the standard error of the mean. (TIF) [file pone.0125356.s002.tif]

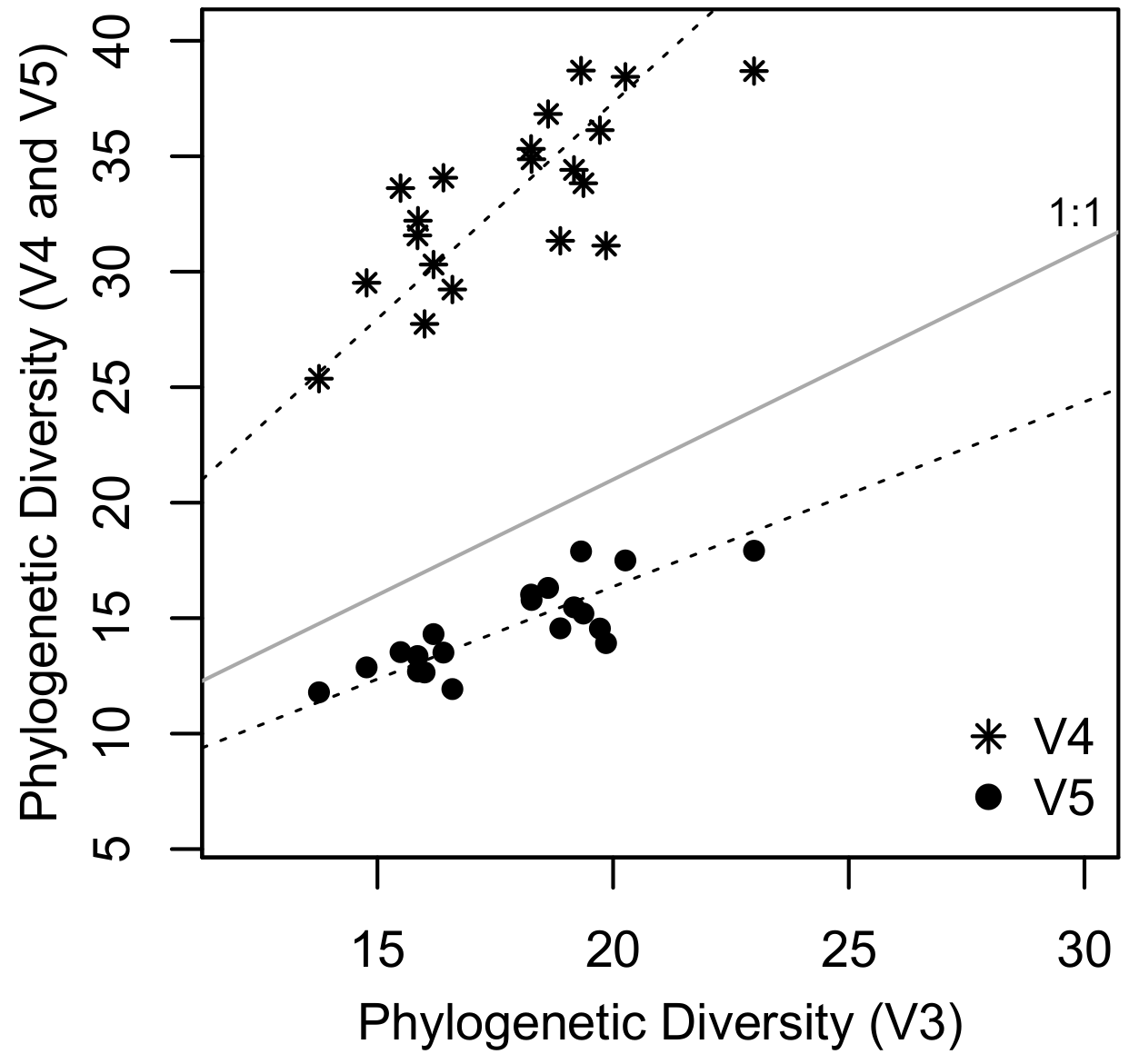

Supplement: S3 Fig — Central line shows 1:1 line, dashed lines show the Major Axis (MA) model regression slopes of the two comparisons. (TIF) [file pone.0125356.s003.tif]

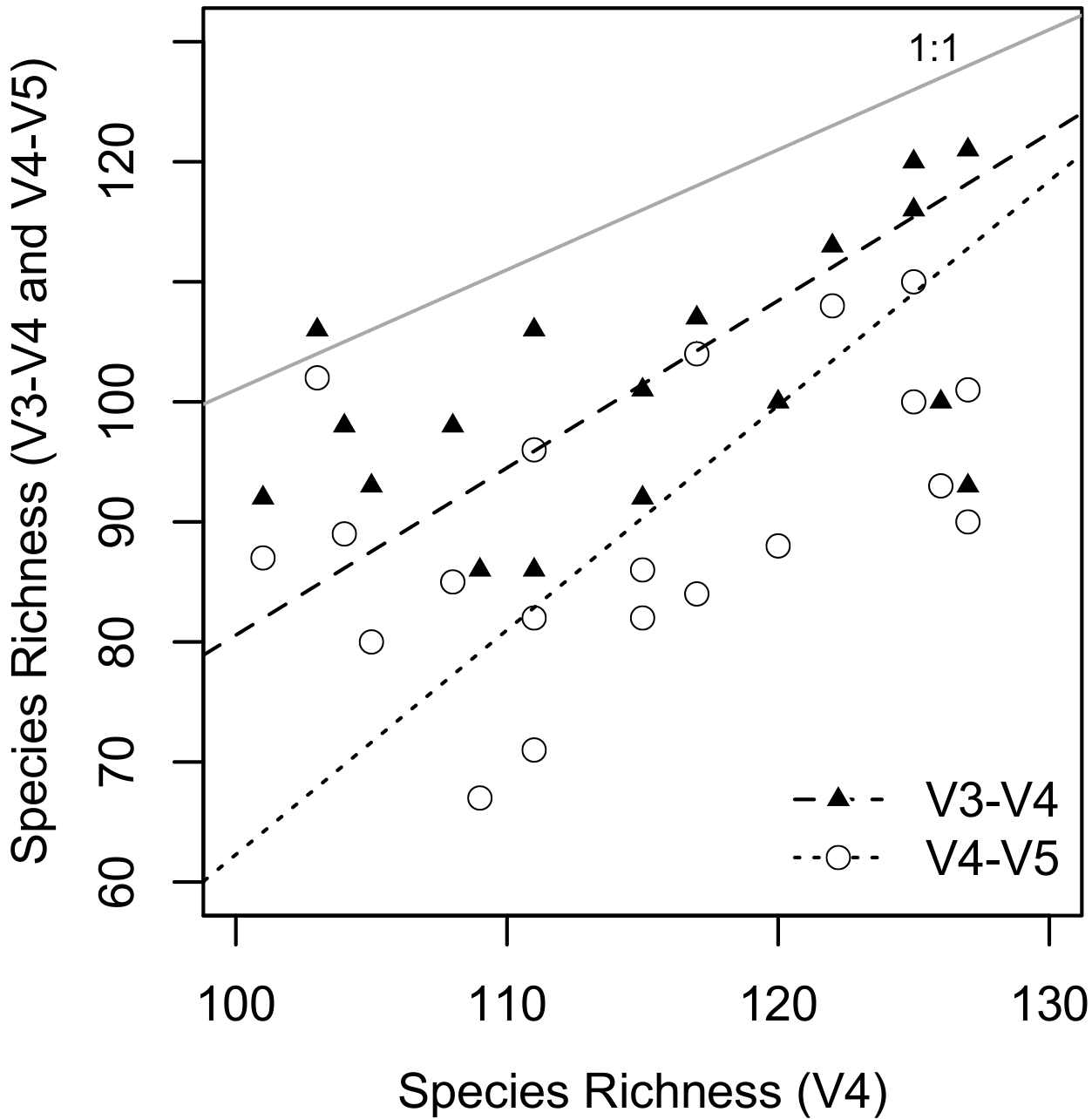

Supplement: S4 Fig — Central line shows 1:1 line, dashed lines show the MA model regression slopes of the comparisons. (TIF) [file pone.0125356.s004.tif]

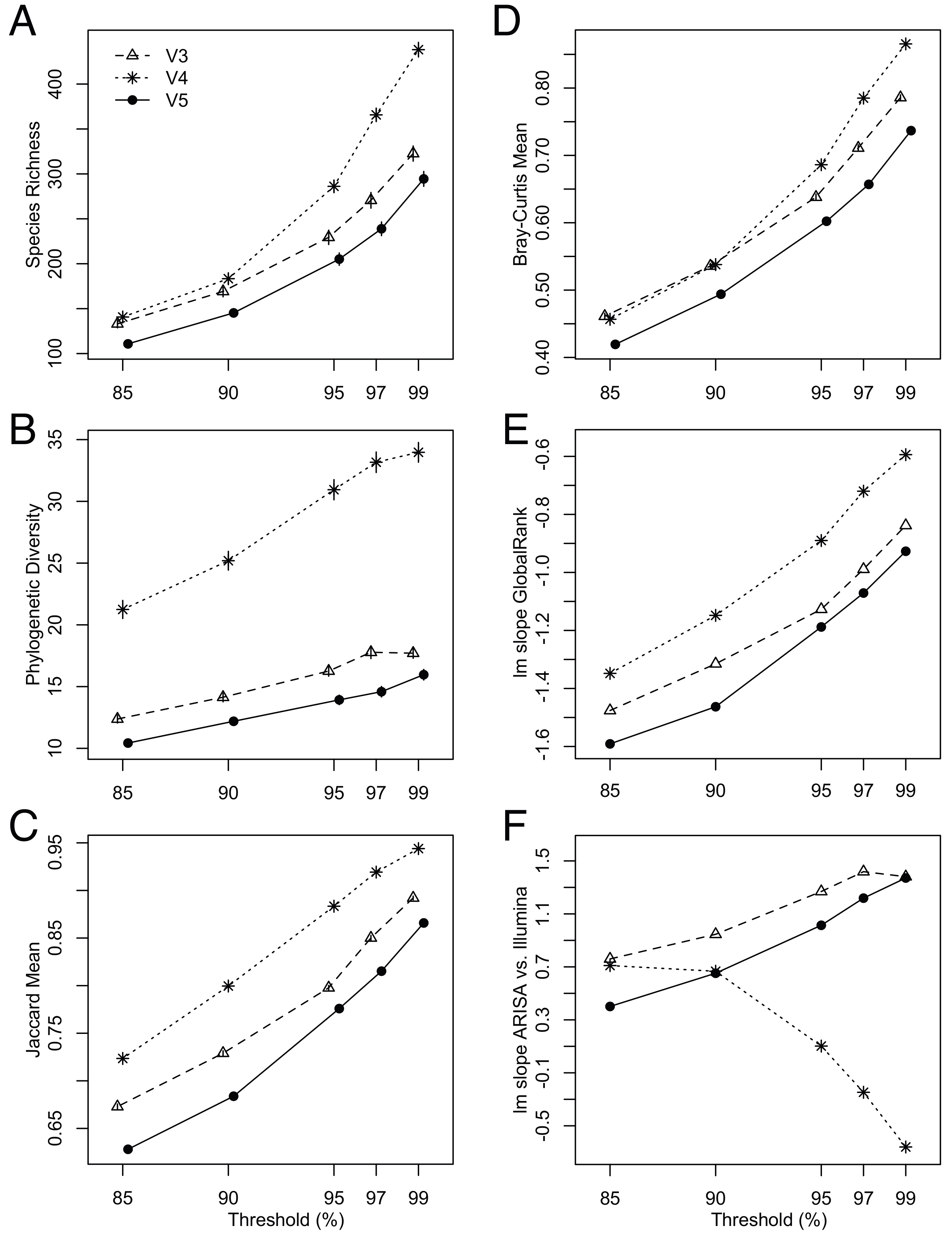

Supplement: S5 Fig — A: changes in SR, B: changes in PD, C: changes in Jaccard dissimilarities, D: changes in Bray-Curtis dissimilarities, E: changes in global rank-abundance slopes, F: changes in the linear model slopes between ARISA Fingerprints and Illumina sequencing. The graph shows the mean and standard error (SE) for each region and SST. (TIF) [file pone.0125356.s005.tif]

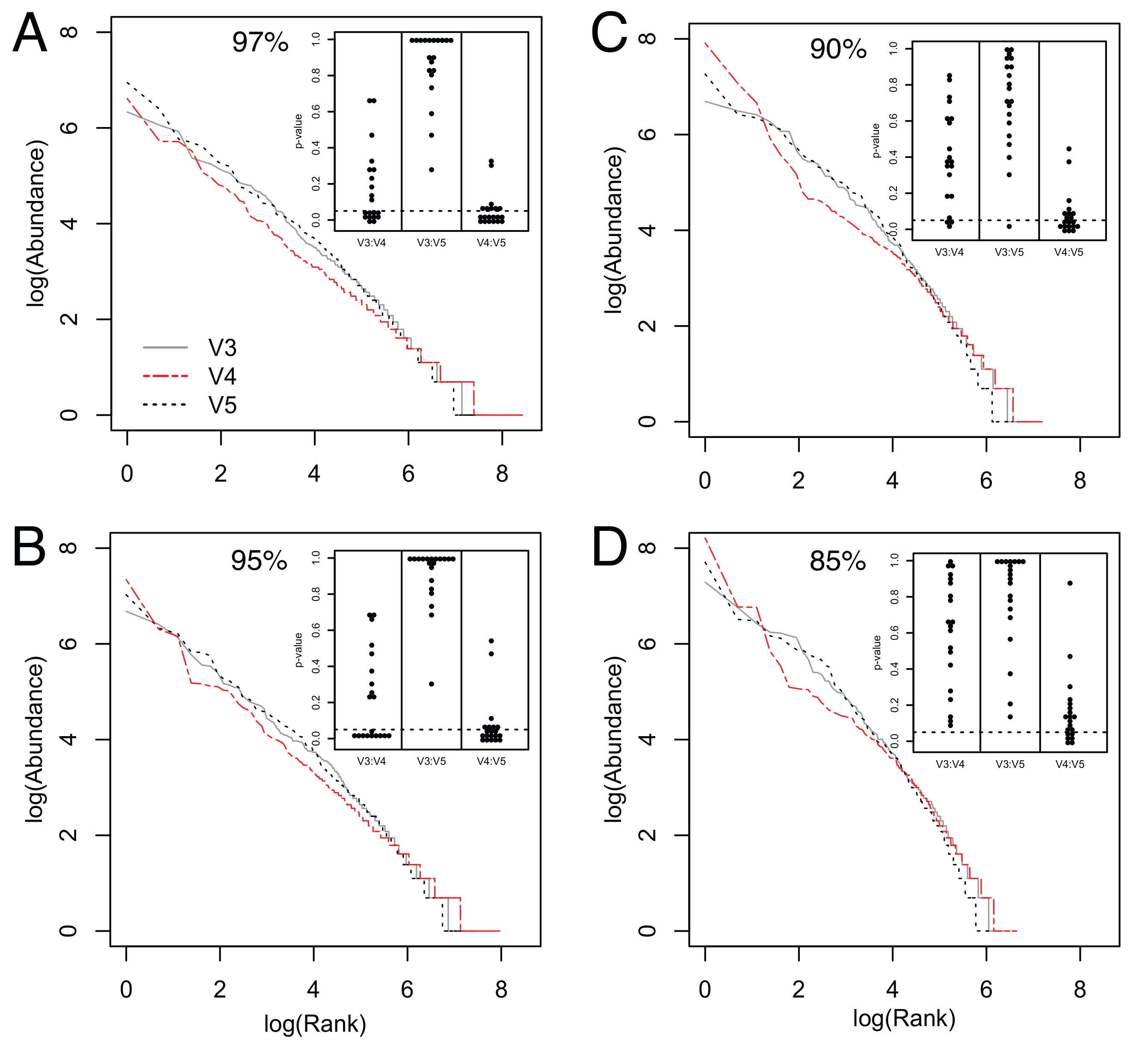

Supplement: S6 Fig — Large plots: Rank plotted against abundance on a log-log scale. Small plots show the changes in significant differences between the three variable regions (see Fig 5C for a more detailed description of the inlay plots). (TIF) [file pone.0125356.s006.tif]

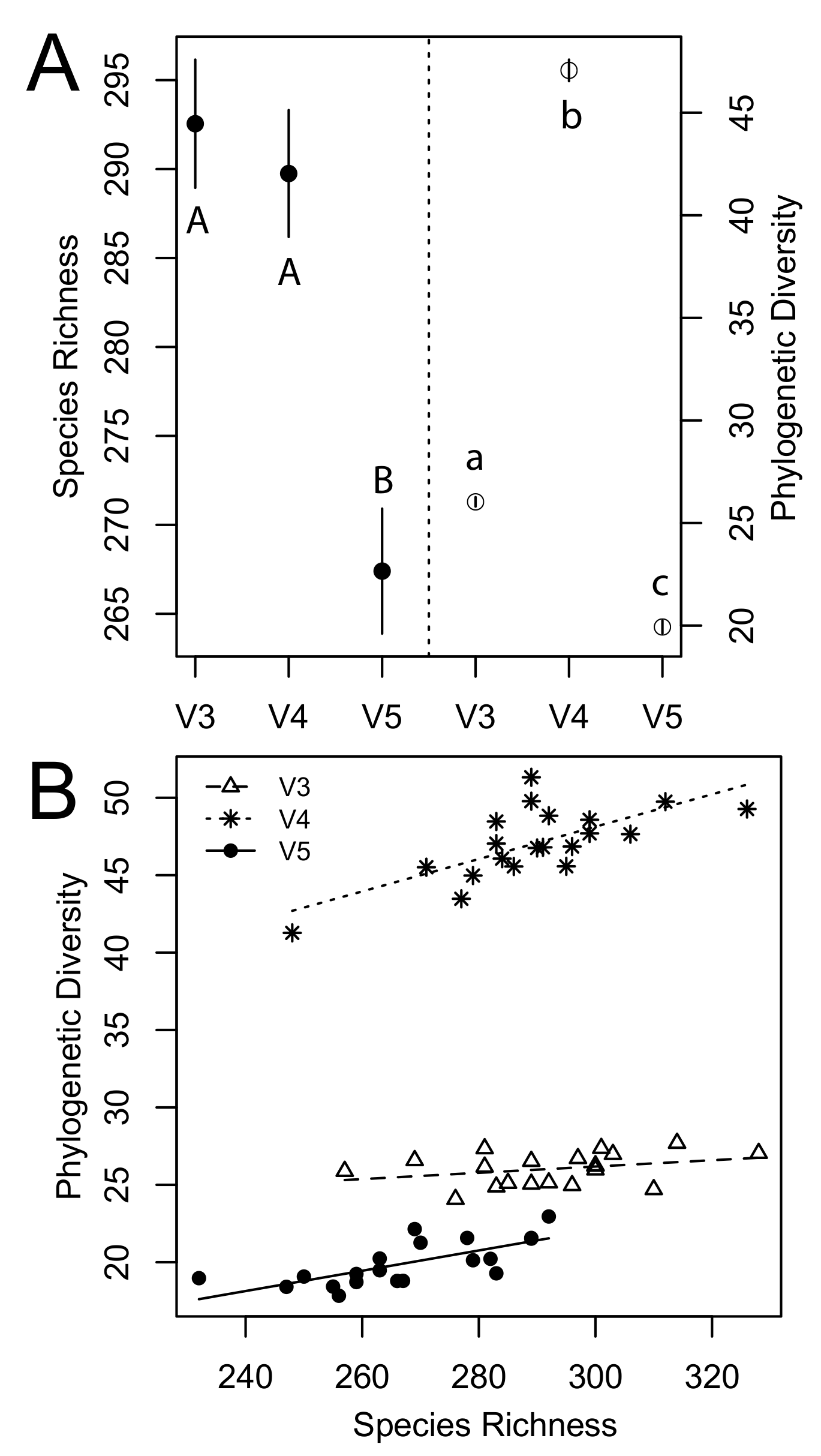

Supplement: S7 Fig — A: Rarefied SR and PD calculated from a subset of the Greengenes database using the same parameters used as for the lake survey data. Points show the mean and lines the standard error of the mean. PD was significantly higher in the V4 dataset (F = 1406, p < 0.001) compared to the V3 and V5 datasets. The SR of the V4 region dataset was only significantly different form the V5, but not from the V3 dataset (F = 14.99, p < 0.001). B: SR plotted against PD for each of the three variable regions where each dot represents one lake sample. Lines show the MA regression model for each dataset. R2 values: V3 = 0.10, V4 = 0.49, V5 = 0.50. (TIF) [file pone.0125356.s007.tif]
