# Supplementary material for: Estimating Bacterial Diversity for Ecological Studies: Methods, Metrics, and Assumptions
Source: PLoS One. 2015 Apr 27;10(4):e0125356. doi: 10.1371/journal.pone.0125356 (PMC4411174; doi:10.1371/journal.pone.0125356)
Supplement: S1 Table — (PDF) [file pone.0125356.s008.pdf]

S1 Table: Number of quality filtered reads of the different lakes and variable regions of the 16S rRNA (untrimmed and trimmed reads).

		Number of reads after quality filtering					
Lake name	Abbreviaton	untrimmed	V3	V4	V5	V3-V4	V4-V5
Baldeggersee	Ba	4809	1211	1561	1065	389	301
Bielersee	Bi	5584	1141	2052	1003	359	315
Brienzersee	Br	7134	1069	2711	926	197	250
Burgäschisee	Bu	5537	1199	1865	1109	365	332
Caumasee	Ca	4344	901	1559	683	184	212
Greifensee	Gr	6302	1207	1614	1384	245	232
Halwilersee	Ha	6337	992	2034	943	192	209
Inkwilersee	In	4256	918	1449	920	243	238
Lag Grand	LG	6116	1011	2162	1405	219	285
Melchsee	Me	7959	1445	3058	1242	240	277
Murtensee	Mu	8461	1634	2872	1565	422	432
Neuenburgersee	Ne	5221	990	1730	974	250	233
Rotsee	Ro	5253	1154	1684	1082	360	330
Sempachersee	Se	3508	754	962	691	237	195
Soppensee	So	5074	959	1757	954	209	229
Thunersee	Th	5455	941	1798	901	268	268
Türlersee	Tu	5124	1081	1594	1059	281	257
Vierwaldstättersee	Vi	5262	1074	1869	969	361	333
Zürichsee	ZS	5569	1113	1288	1184	222	189
Zugersee	Zu	5557	995	1804	844	218	197
Total # of reads		112917	21798	37432	20909	5461	5315
Mean # of reads (Standard deviation)		5643 (1155)	1089	1871	1045	273	266
