# Supplementary material for: Estimating Bacterial Diversity for Ecological Studies: Methods, Metrics, and Assumptions
Source: PLoS One. 2015 Apr 27;10(4):e0125356. doi: 10.1371/journal.pone.0125356 (PMC4411174; doi:10.1371/journal.pone.0125356)
Supplement: S2 Table — Positions are given relative to E. coli 16S rRNA positions. (PDF) [file pone.0125356.s009.pdf]

S2 Table: Positions of 11-mers used for subsetting the quality filtered Illumina reads as well as the number of nucleotides (nt) of the trimmed datasets. Positions are given relative to E. coli 16S rRNA positions.

16S region	11-mer position	reading direction	# of nt
V3	518-508	reverse	120
V4	558-568	forward	120
V5	779-789	forward	100
V3-V4	415-425	forward	460
V4-V5	789-779	reverse	310
