# Supplementary material for: Estimating Bacterial Diversity for Ecological Studies: Methods, Metrics, and Assumptions
Source: PLoS One. 2015 Apr 27;10(4):e0125356. doi: 10.1371/journal.pone.0125356 (PMC4411174; doi:10.1371/journal.pone.0125356)
Supplement: S3 Table — (PDF) [file pone.0125356.s010.pdf]

S3 Table: Environmental parameters of the lakes that were sampled for this study.

Lake	Sampling date	Sampling location [Lat, Long]	maximum Depth [m]	Elevation [m]	DOC [mg C/L]	NO_3 [mg/L]	PO_4 [μg/L]	Temp [°C]	pH	Oxygen [mg/L]	Chl a [mg/L]	Secchi depth [m]
Baldeggersee (Ba)	10-10-2011	47.0509 N 7.1026 E	66	463	4.0	0.8	0.5	15.82	7.97	9.63	32.13	1.5
Bielersee (Bi)	14-09-2011	46.4340 N 7.5757 E	74	429	1.7	0.6	1.4	20.09	8.12	9.35	3.12	4.5
Brienzersee (Br)	12-07-2011	47.1008 N 7.4004 E	261	564	1.7	0.3	4.4	17.83	8.27	10.00	0.96	3.0
Burgaschisee (Bu)	05-10-2011	46.4911 N 9.1745 E	31	465	7.6	0.7	1.3	19.38	8.35	9.74	6.06	4.0
Caumasee (Ca)	19-07-2011	47.2110 N 8.4037 E	30	997	1.2	0.3	1.0	16.90	8.58	8.81	1.64	7.0
Greifensee (Gr)	15-07-2011	47.1749 N 8.1239 E	32	435	4.4	1.5	1.9	21.90	8.35	11.41	9.98	2.5
Halwilersee (Ha)	13-07-2011	47.1152 N 7.3943 E	47	449	8.5	0.6	4.2	23.06	8.30	9.83	1.74	6.0
Inkwilersee (In)	05-10-2011	46.4828 N 9.1525 E	6	461	7.5	0.3	2.1	17.75	7.21	4.21	37.46	1.5
Lag Grond (LG)	19-07-2011	46.4622 N 8.1622 E	5	1016	2.8	0.3	1.0	17.41	7.80	6.26	11.15	1.5
Melchsee (Me)	12-07-2011	46.5555 N 7.0506 E	18	1891	2.9	0.3	4.4	17.58	8.00	8.71	2.82	4.0
Murtensee (Mu)	15-09-2011	46.5421 N 6.5132 E	45	429	3.8	1.4	1.7	20.73	8.20	9.46	10.50	2.0
Neuenburgersee (Ne)	14-09-2011	47.0227 N 8.1106 E	153	429	2.6	0.7	1.2	21.10	7.97	9.40	2.26	7.0
Rotsee (Ro)	06-10-2011	47.1138 N 8.2110 E	16	419	1.8	0.3	2.3	18.71	8.61	12.76	8.37	3.0
Sempachersee (Se)	17-10-2011	47.0838 N 8.0907 E	87	505	3.8	0.3	1.0	15.31	8.31	8.55	4.42	4.5
Soppensee (So)	18-07-2011	47.0523 N 8.0451 E	28	596	6.4	0.9	1.2	21.00	8.49	11.42	6.15	3.5
Thunersee (Th)	11-07-2011	46.4246 N 7.4022 E	217	558	1.1	0.4	3.2	18.27	8.39	9.66	0.71	6.5
Türlersee (Tu)	20-07-2011	47.1612 N 8.3010 E	22	643	3.8	0.3	1.0	19.85	8.45	11.77	12.14	3.0
Vierwaldstättersee (Vi)	14-10-2011	47.0108 N 8.2403 E	214	434	1.2	0.4	1.0	14.75	8.15	9.50	2.62	6.2
Zugersee (Zu)	14-07-2011	47.0939 N 8.2858 E	198	413	6.9	0.3	6.0	22.42	8.11	9.75	4.06	3.0
Zürichsee (ZS)	22-07-2011	47.1734 N 8.3437 E	136	407	1.6	0.5	1.0	19.75	8.25	10.23	4.46	3.0
