# Supplementary material for: Estimating Bacterial Diversity for Ecological Studies: Methods, Metrics, and Assumptions
Source: PLoS One. 2015 Apr 27;10(4):e0125356. doi: 10.1371/journal.pone.0125356 (PMC4411174; doi:10.1371/journal.pone.0125356)
Supplement: S4 Table — (PDF) [file pone.0125356.s011.pdf]

S4 Table: Linear model statistics of species richness (SR; ARISA and Illumina sequencing) and phylogenetic diversity (PD) versus environmental parameter.

Parameter	ARISA slope (p-value)	V3 SR s (p)	V4 SR s (p)	V5 SR s (p)	SR F-value (p)	V3 PD s (p)	V4 PD s (p)	V5 PD s (p)	PD F-value (p)
MaxDepth	0.02 (0.51)	-7.18 (0.37)	-1.85 (0.79)	-1.51 (0.83)	0.20 (0.82)	-0.13 (0.78)	-1.02 (0.17)	-0.18 (0.65)	0.88 (0.42)
Elevation	-0.01 (0.14)	-21.97 (0.35)	18.39 (0.36)	-8.19 (0.69)	0.97 (0.39)	-0.51 (0.71)	-0.31 (0.89)	-0.42 (0.71)	0.00 (1.00)
DOC	-0.54 (0.65)	0.45 (0.98)	-13.75 (0.24)	-11.29 (0.35)	0.38 (0.69)	-0.55 (0.50)	-0.22 (0.87)	-0.43 (0.53)	0.03 (0.97)
NO_3	-2.79 (0.72)	27.54 (0.10)	21.87 (0.13)	20.75 (0.16)	0.06 (0.94)	0.88 (0.38)	2.45 (0.12)	0.89 (0.28)	0.64 (0.53)
PO_4	-0.83 (0.66)	-28.79 (0.03)	-7.92 (0.51)	-22.46 (0.05)	0.85 (0.43)	-0.40 (0.62)	-2.59 (0.04)	-1.16 (0.08)	1.56 (0.22)
Temp	-1.22 (0.31)	-73.22 (0.32)	-24.78 (0.70)	-87.46 (0.16)	0.26 (0.77)	-1.14 (0.79)	-6.27 (0.37)	-4.52 (0.20)	0.27 (0.76)
pH	-1.55 (0.87)	66.21 (0.78)	160.57 (0.42)	140.00 (0.49)	0.06 (0.95)	10.44 (0.44)	12.76 (0.57)	11.23 (0.32)	0.01 (1.00)
DO	0.10 (0.95)	34.11 (0.38)	39.69 (0.23)	39.54 (0.24)	0.01 (0.99)	3.84 (0.08)	3.29 (0.37)	2.62 (0.16)	0.05 (0.95)
Chla	0.26 (0.37)	20.54 (0.01)	-0.58 (0.94)	9.39 (0.22)	2.04 (0.14)	0.50 (0.33)	1.80 (0.02)	0.56 (0.18)	1.75 (0.18)
Secchi	-0.85 (0.58)	-24.48 (0.18)	7.31 (0.64)	-4.81 (0.77)	0.97 (0.39)	-0.27 (0.81)	-1.88 (0.28)	-0.33 (0.71)	0.53 (0.59)
