# Supplementary material for: Estimating Bacterial Diversity for Ecological Studies: Methods, Metrics, and Assumptions
Source: PLoS One. 2015 Apr 27;10(4):e0125356. doi: 10.1371/journal.pone.0125356 (PMC4411174; doi:10.1371/journal.pone.0125356)
Supplement: S5 Table — (PDF) [file pone.0125356.s012.pdf]

S5 Table: Linear model statistics of species richness (SR) estimates from ARISA and Illumina sequencing at different sequence similarity threshold (SST) clustering values.

16S region	SST	slope	R^2	F-value	p-value
V3	85	0.76	0.20	4.59	0.05
V3	90	0.95	0.18	3.85	0.07
V3	95	1.27	0.18	3.81	0.07
**V3**	**97**	1.42	0.18	3.99	0.06
V3	99	1.38	0.19	4.09	0.06
V4	85	0.71	0.12	2.54	0.13
V4	90	0.67	0.08	1.66	0.21
V4	95	0.10	0.00	0.03	0.87
**V4**	**97**	-0.25	0.01	0.14	0.72
V4	99	-0.66	0.05	0.86	0.37
V5	85	0.40	0.09	1.85	0.19
V5	90	0.65	0.13	2.68	0.12
V5	95	1.01	0.15	3.21	0.09
**V5**	**97**	1.22	0.18	3.89	0.06
V5	99	1.37	0.19	4.24	0.05
