# Supplementary material for: Estimating Bacterial Diversity for Ecological Studies: Methods, Metrics, and Assumptions
Source: PLoS One. 2015 Apr 27;10(4):e0125356. doi: 10.1371/journal.pone.0125356 (PMC4411174; doi:10.1371/journal.pone.0125356)
Supplement: S6 Table — (PDF) [file pone.0125356.s013.pdf]

S6 Table: Paired t-test results of the ten most abundant bacterial classes.

Bacterial class*	global	**V3 / V4**		**V3 / V5**		**V4 / V5**	
	Abundance [%]	T-statistic	p-value	T-statistic	p-value	T-statistic	p-value
Betaproteobacteria	33.16	-12.58	<0.01	10.81	<0.01	19.19	<0.01
Alphaproteobacteria	8.53	1.14	0.27	-3.48	<0.01	-3.20	<0.01
Gammaproteobacteria	5.66	2.99	<0.01	3.23	<0.01	1.19	0.25
Synechococcophycideae	5.33	-4.54	<0.01	-1.63	0.12	3.39	<0.01
Flavobacteriia	5.16	10.08	<0.01	2.82	0.01	-13.24	<0.01
[Saprospirae]*	4.42	13.65	<0.01	-2.98	<0.01	-12.16	<0.01
Chloroplasts	3.90	-0.42	0.68	-4.97	<0.01	-3.10	<0.01
Deltaproteobacteria	1.78	-3.62	<0.01	-2.72	0.01	1.68	0.11
[Methylacidiphilae]*	1.64	3.66	<0.01	-2.14	0.05	-3.24	<0.01
Sphingobacteriia	1.59	8.68	<0.01	0.75	0.47	-7.50	<0.01

**Note:* Data was calculated for the ten most abundant classes across the complete dataset. Square brackets indicate candidate class names.
